# Acute home-based management initiatives for COVID-19 patients needing oxygen: observational study reporting mortality and hospital admission outcomes

**DOI:** 10.1186/s12913-025-13733-2

**Published:** 2025-11-25

**Authors:** Josi A. Boeijen, Alma C. van de Pol, Abeer Ahmad, Martijn R. Mantingh, Eric van Rijswijk, Marjan J. van Apeldoorn, Eric van Thiel, Netty de Graaf, Florian Heesbeen, Roderick P. Venekamp, Frans H. Rutten, Dorien L. M. Zwart

**Affiliations:** 1 Department of General Practice & Nursing Science, Julius Center for Health Sciences and Primary Care, University Medical Center Utrecht, Utrecht University, Utrecht, The Netherlands; 2 Regional Organization for General Practice Drenthe, Dokter Drenthe, Assen, The Netherlands; 3 Primary Care Network Jeroen Bosch Huisartsen, Vught, The Netherlands; 4 Department of Internal Medicine, Jeroen Bosch Hospital, ’s-Hertogenbosch, The Netherlands; 5 Department of Pulmonology, Albert Schweitzer Hospital, Dordrecht, The Netherlands

**Keywords:** COVID-19, Oxygen therapy, Telemedicine, Acute home-based care, Hospital at home, Collaborative care

## Abstract

**Objective:**

Acute home-based management for COVID-19 patients requiring oxygen therapy was introduced during the pandemic as an alternative to in-hospital care and early hospital discharge. This study aims to synthesize clinical course data, health care resource use data, and mortality rates of patients who received acute home-based management during the pandemic in the Netherlands.

**Method:**

Retrospective cohort study of patients receiving acute home-based management including oxygen therapy for COVID-19 across five Dutch regional initiatives between December 2020 and January 2022. The general practitioner (GP) provided medical care in three initiatives, whereas two initiatives were hospital-led.

**Results:**

Data were collected for 92 patients (mean age 63 [standard deviation (SD) 13] years, 54% male). The median number of days of oxygen therapy and remote monitoring were 8.5 (IQR: 6-13) and 14 (IQR: 9-18), respectively. In total, 17 patients (18%) required hospital admission following acute home-based management, of whom six died during admission. No patients died at home. A median of 1 (interquartile rage [IQR]: 0-4) GP consultation took place per patient during home-based management; 64% via phone and 31% home visits. In hospital-led initiatives, the GP was consulted at least once in 46% of patients.

**Conclusion:**

Acute home-based management of COVID-19 patients requiring oxygen therapy resulted in avoidance of hospital admission in the majority of patients, however at the price of a higher workload for GPs.

**Supplementary Information:**

The online version contains supplementary material available at 10.1186/s12913-025-13733-2.

## Introduction

During the COVID-19 pandemic, several initiatives were developed to manage patients at home using telemonitoring and supplemental oxygen therapy via nasal cannula. Most of these initiatives focussed on early discharge of initially admitted patients and reported promising results [1–6]. Yet, reports on *acute* home-based care programs, i.e. without initial hospital admission, are scarce.

Acute home-based instead of hospital-based management of COVID-19 patients may benefit patients [7–9] and support their preference to remain at home. It may also alleviate the significant pressure on hospital care associated with acute respiratory tract infections [5, 10, 11]. Moreover, acute home-based management has the potential to substantially reduce healthcare costs by avoiding hospital care [2, 12–15].

We previously described the design of five Dutch acute home-based management initiatives for COVID-19 patients needing oxygen therapy [16]. All these initiatives had a collaborative care approach centred around the patient staying at home and included remote monitoring of patients’ vital signs and wellbeing (i.e., oxygen saturation, heart rate, severity of shortness of breath on a visual analogue scale and sometimes breathing rate and blood pressure). To date, clinical outcome data such as re-admission and mortality rates of acute home-based management initiatives are largely unknown. Several retrospective, observational studies have been reported which describe low hospital admission and mortality rates for similar initiatives in other countries, albeit in patients who were less severely ill and often did not need oxygen therapy at home [10, 17–23].

This study aimed to synthesize clinical course data, healthcare resource use data, and mortality rates from patients who received acute home-based management during the COVID-19 pandemic in the Netherlands.

## Method

### Design and setting

We conducted a retrospective cohort study of patients receiving acute home-based management for COVID-19 between December 2020 and January 2022 across five collaborative care initiatives in the Netherlands (Supplementary Table 1).

The five initiatives were previously selected and described in design [16]. In short, initiatives were selected if they provided COVID-19 patients (i) acute home-based management (ii) supplemental oxygen treatment at home, (iii) structured remote monitoring of at least peripheral oxygen saturation (SpO_2_). Remote monitoring was either provided via an app (telemonitoring) or via phone contact. Medical care was either coordinated by the patient’s own general practitioner (GP) or by a hospital physician; the collaborative care organisation differed across the initiatives. Of the five initiatives, four included a protocolled home visit by a home care nurse or GP [16].

### Study population

Patients participating in one of these five initiatives were eligible. Patients who were admitted to the hospital for longer than 24 h prior to entering the home-based management were excluded. Brief (< 24 h) hospital or emergency department (ED) admission was sometimes necessary for logistical set-up of home-based care. Consequently, patients who were admitted to the hospital for shorter than 24 h were not excluded and thus considered acute home-based managed patients.

**Fig. 1:**
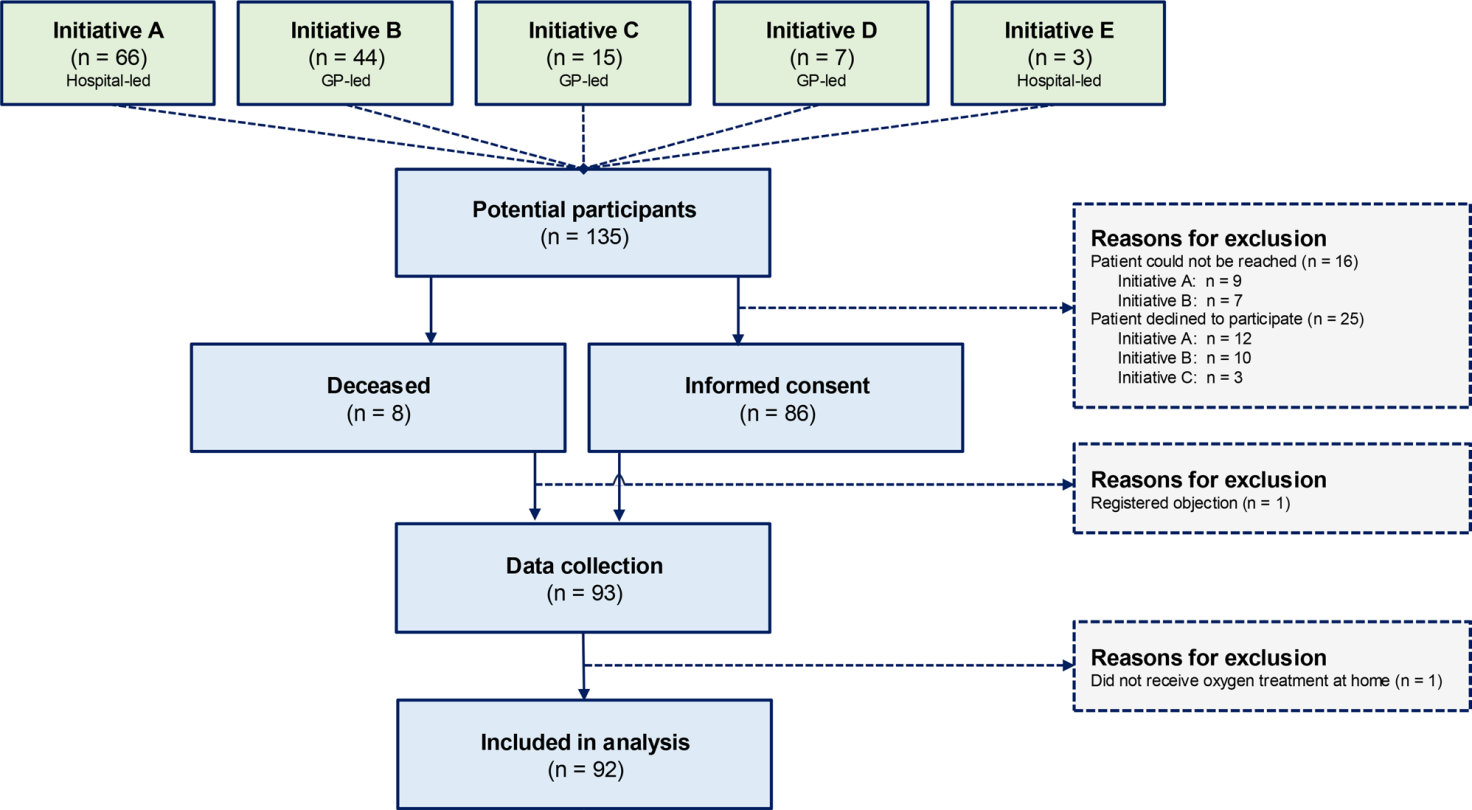
Participant flow-diagram

Target populations varied per initiative. All patients had mild hypoxaemia, but the definition varied substantially; SpO2 93% or higher with a maximum of four litres of supplemental oxygen per minute; SpO2 90% or higher without oxygen supplementation; SpO2 93% or lower and/or respiratory rate >24 per minute; or based on arterial blood gas measurement (PaO_2_ of 60–79 mmHg but no other signs of respiratory failure such as pCO_2_ > 6.65 kPa and pH < 7.35). Two Initiatives (C and D, see Fig. 1) explicitly aimed at acutely managing frail patients in the home setting. The other three initiatives in contrast tailored the home intervention to patients in whom an uncomplicated disease trajectory was expected [16].

### Informed consent procedure

Patients gave written informed consent for collection of their data. For deceased patients, data was collected unless the patient had registered a general objection for research participation.

### Data collection

Patient’s hospital charts and GP electronic health records were reviewed to identify patient characteristics (age, gender, height, weight) and the presence of any of the following comorbidities: diabetes mellitus type 1 and 2, Chronic Obstructive Pulmonary Disease (COPD), asthma, heart failure, coronary artery disease (coronary artery bypass grafting (CABG), myocardial infarction (MI) or percutaneous coronary intervention (PCI)), atrial fibrillation, stroke or Transient Ischemic Attack (TIA), chronic kidney failure, and immunodeficiency. Clinical course data were collected by reviewing the remote monitoring data registered in the telemonitoring system (Luscii Healthtech application [24]) or telephone follow-up registration forms from the monitoring centre. Data on health care resource use were obtained from hospital and ED charts, and GP electronic health records. Mortality data were obtained from hospital charts and GP health records.

### Data analysis

Patient characteristics and outcomes were reported descriptively. Dichotomous variables were presented as proportions and continuous variables as means with standard deviations (SDs) when normally distributed or as medians and interquartile ranges (IQR) when abnormally distributed. We did not impute missing data. Data were analysed using IBM SPSS 27.0 and Microsoft Excel.

## Results

The process of obtaining informed consent and collecting patient data took place between August 2022 and May 2023. Initially, 135 patients were identified as eligible (Fig. 1). Data were available for 93 patients (69%); 25 (19%) declined participation, and 16 (12%) could not be reached. During data collection, one patient was excluded because the home-based management did not include supplemental oxygen therapy.

Baseline characteristics of included patients are depicted in Table 1 and Supplementary Table 2. Of the 92 included patients the mean age was 63 years (SD 13) and 54% were male. Most patients (52%) did not have comorbidities (diabetes mellitus, COPD, asthma, heart failure, coronary artery disease, atrial fibrillation, stroke/TIA, chronic kidney failure, or immunodeficiency), whereas 24% had more than two comorbidities.

**Table 1:** Patient characteristics of the 92 participants receiving acute home-based care

Characteristic [missing]	Total *n* = 92
Male gender, n (%) [0]	50 (54)
Age in years, mean (SD) [0]	63 (13)
Age in years, range [0]	26–96
BMI ≥30 kg/m2, n (%) [29]	21 (33)
Current cigarette smoking, n (%) [14]	5 (6)
Any comorbidity, n (%) [3]	43 (48)
≥2 comorbidities, n (%) [4]	21 (24)
**Comorbidities**	
Diabetes, n (%) [2]	14 (16)
COPD, n (%) [2]	11 (12)
Asthma, n (%) [2]	9 (10)
Heart failure, n (%) [3]	3 (3)
Coronary disease (PCI, CABG, or MI), n (%) [2]	13 (14)
Atrial fibrillation, n (%) [2]	7 (8)
Stroke/TIA, n (%) [2]	10 (11)
Chronic kidney failure, n (%) [3]	5 (6)
Immunodeficiency, n (%) [2]	3 (3)

Abbreviations: BMI = body mass index; CABG = coronary artery bypass grafting; COPD = chronic obstructive pulmonary disease; MI = myocardial infarction; PCI = percutaneous coronary intervention; TIA = transient ischemic attack

### Clinical course data

Patients had COVID-19-related symptoms for a median of 9 (IQR: 6–11) days before the start of acute home-based management. The initial SpO_2_ (without oxygen) was < 88% in 8%, < 91% in 27%, and < 95% in 67% of patients. The amount of oxygen administered was ≤ 2.0 L/min in 45%, and 5.0 L/min (the maximum) in 9% of patients.

The median number of days of oxygen therapy and monitoring were 8.5 (IQR: 6–13) and 14 (IQR: 9–18), respectively (Table 2), with notable differences between initiatives (Supplementary Table 3).

**Table 2:** Clinical course data, hospital admissions and mortality

Parameter [missing]	Total *n* = 92
Home monitoring (days), median, IQR [1]	14, 9–18
Home oxygen suppletion (days), median, IQR [12]	8.5, 6–13
Hospital admissions (ward or ICU), n (%) [2]	17 (19)
30-day mortality, n (%) [1]	6 (7)

Abbreviations: ICU = intensive care unit; IQR = interquartile range

### Hospital admission and mortality

**Fig. 2:**
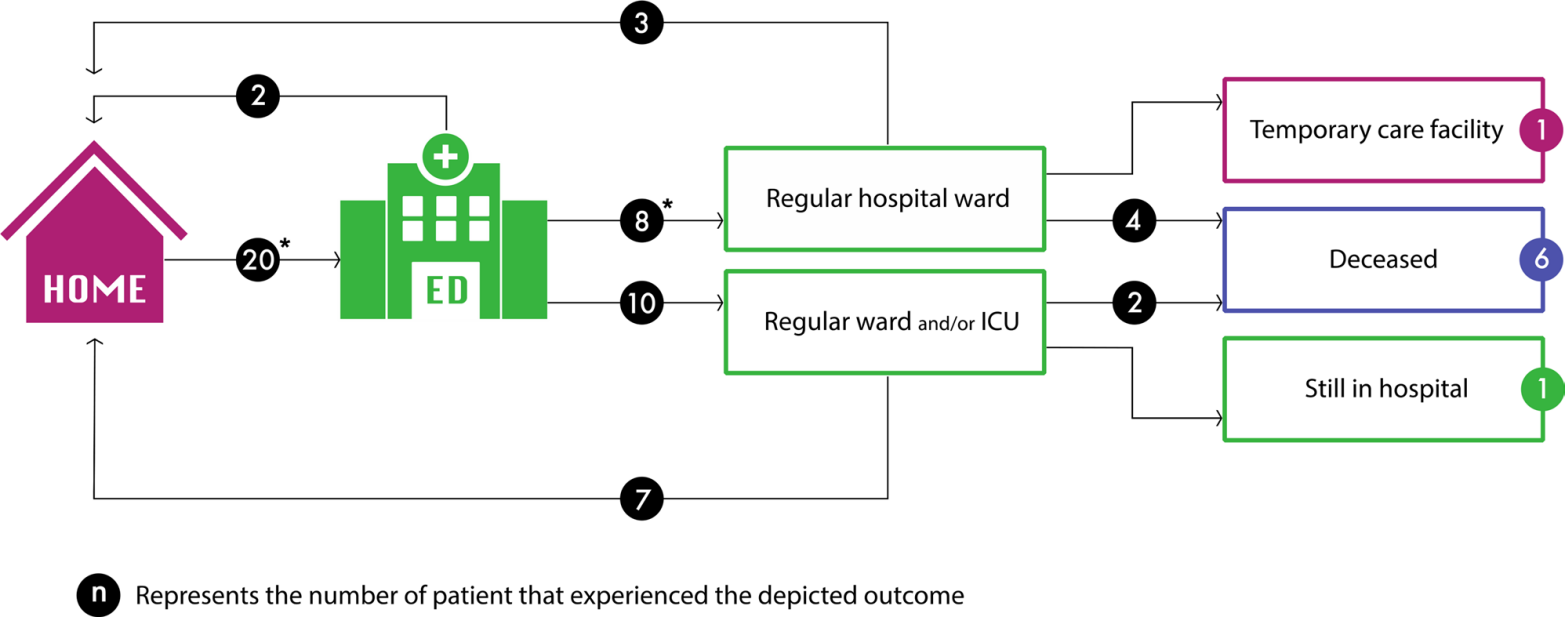
* One patient was presented to the ED and admitted twice. Abbreviations: ED = emergency department; ICU = intensive care unit

Twenty ED presentations were needed in 19 patients (21%) receiving acute home-based management. This resulted in 18 hospital admissions for 17 unique patients (18%), while two patients could directly be discharged from the ED to their home (Fig. 2). One patient was admitted twice, with home-based management in-between both hospital admissions. After 30-days follow-up, one patient was still in the general ward and one patient was discharged to a temporary primary care facility for recovery.

Of the 6 reported deaths (7%), none occurred at home. Two patients died after admission to the intensive care unit, four at the general (COVID-19-)ward. Cause of death was respiratory failure as a result of COVID-19 in 5 patients; one patient died from septic shock. The mortality rate among patients in initiatives A/B/E (aimed at low risk patients) was 4%; the mortality rate in initiatives C/D (aimed at more frail patients) was 18%.

### GP consultations

Data regarding GP consultations during follow-up were available for 78 patients. GP consultation occurred in 52 patients (67%), with a total of 173 consultations. There was a median of 1 (IQR: 0–4) GP consultation per patient. In hospital-led initiatives (A and E), the GP was consulted at least once in 46% of patients (Table 3). The median number of consultations for patients who contacted their GP in a hospital-led initiative was 1 (IQR: 1-3.5). In GP-led initiatives (B, C and D), the median number of GP consultations was 3 per patient (IQR 1–5).

**Table 3:** GP consultations during follow-up

	Total (*n* = 78)	Hospital-led (*n* = 39)	GP-led (*n* = 39)
Total number of GP consultations, n	173	130	43
Number of consultations per patient, median (IQR)	1 (0–4)	0 (0–1)	3 (1–5)
Number of patients with at least 1 consultation, n (%)	52 (67%)	18 (46%)	34 (87%)
Number of GP consultations in patients with at least 1 GP consultations, median (IQR)	2.5 (1–5)	1 (1-3.5)	3 (2–5)

Abbreviations: GP = general practitioner; IQR = interquartile range

Almost half of the consultations (46%) were scheduled (routine). Clinical deterioration and/or abnormal monitoring parameters were among the reasons for consultation in 25% of consultations (non-routine). Other reasons for GP consultation were dysregulation of blood glucose levels, a patient or informal caregiver needing reassurance, social (non-medical) reasons and post-recovery care.

Most consultations were done via phone (64%). Home visits were done in 29 patients, making up 31% of the total number of GP consultations. Other consultations occurred in the GP practice during regular consultation hours (4%); via video calls (1%); or via e-mail (1%). Most consultations (89%) took place during office hours.

## Discussion

### Summary

We synthesized clinical course data, healthcare resource use data, and mortality rates from patients who received acute home-based management during the COVID-19 pandemic in the Netherlands.

The median number of days of oxygen therapy and monitoring were 8.5 (IQR: 6–13) and 14 (IQR: 9–18), respectively. Home-based management resulted in avoidance of hospital admission in 82%. A median of 1 (IQR: 0–4) GP consultations per patient took place. Six deaths occurred in hospital (7%), none of the patients died at home.

### Comparison with existing literature

Schoenling et al. retrospectively report on the outcomes of 632 COVID-19 patients who were managed at home with oxygen therapy after presentation at the ED of 14 different hospitals in the US. Patients were provided with a pulse oximeter and return guidelines provided. Primary care physicians or other healthcare professionals assumed follow after discharge; telemonitoring was optional and not available in all hospitals. Similar to our reported data, 75% of patients in this cohort recovered outside the hospital; 24% was hospitalized. The cohort’s overall mortality rate was 8%, with 7% of patients dying in hospital and 0.5% outside the hospital.

Several previous studies about hospitalized COVID-19 patients reported on average a mortality rate of around 20% [25, 26]. The case mix of patients in our study, however, precludes comparison with data from hospitalised populations.

Previous studies in hospitalized COVID-19 patients reported an average length of stay of 6–15 days [25–28]. In our study, patients needed a median of 8.5 days oxygen and 14 days monitoring.

Two previous studies reported on the mean number of days of oxygen treatment and length of remote monitoring in home-based management programs [23, 29]. These US and France-based studies described combined results of patients who were (i) early discharged after hospital admission and (ii) acutely managed at home. The US-study reported on 621 COVID-19 patients, 24% of which were managed acutely at home after ED evaluation [23]. The France-based study reported on 300 patients, with an unknown minority of patients managed acutely at home [29]. Data were not presented separately for these strategies, which hampers direct comparison with our results.

### Strengths and limitations

We were able to include all Dutch acute home-based care initiatives and scrutinized all available routine care data to minimize missing values.

Some limitations should be noted. We performed an observational study and thus had no (randomised) control group for comparison. As a result, it is not possible to directly compare acute home-based management to conventional hospital treatment and thus cannot conclude that acute home-based management is ‘equivalent’, ‘better’ or ‘safer’ than conventional hospital care for COVID-19 patients needing oxygen therapy.

Interpretating our data, one should realize that the five initiatives were heterogeneous because two explicitly included (more) frail patients while the others focused on lower risk patients. Finally, we could not compare our study directly to others because these were lacking or at least not published.

### Implications for practice and future research

Beyond the COVID-19 pandemic, other acute respiratory tract conditions could be considered for acute home-based management, e.g. influenza, bacterial pneumonia and exacerbations of COPD. Acute home-based management initiatives can be organized more easily in countries with strong primary care, and with good communication between hospital specialists and GPs. Acute home-based management may alleviate the overall healthcare burden, lowering hospital care dramatically, however at the price of a higher workload for GPs. Acknowledging that GPs have a negligible workload for hospitalized patients, our data indicate that the workload for GPs would indeed increase. In hospital-led initiatives, the GP was consulted at least once in 46% of patients. The number of GP consultations increased to three consultations per patient in GP-led initiatives.

Previous studies already reported positive patient experiences with remote monitoring [30–32], but a formal assessment of safety and cost-saving potential of acute home-based management of patients with supplemental oxygen requirement caused by an acute respiratory tract infection is still needed. Therefore, a randomised clinical trial comparing such care to hospital management is warranted.

## Conclusions

Acute home-based management of COVID-19 patients requiring oxygen therapy resulted in avoidance of hospital admission in the vast majority of patients, however at the price of a higher workload for GPs. If proven safe and cost-effective, acute home-based management initiatives could also be applied to patients with other acute respiratory tract conditions and thus hold promise to alleviate the overall healthcare burden in these populations as well.

## Supplementary Information

Below is the link to the electronic supplementary material.

Supplementary Material 1

## Data Availability

The datasets used and/or analysed during the current study are available from the corresponding author on reasonable request.

## Abbreviations

BMI: Body mass index
CABG: Coronary artery bypass grafting
COPD: Chronic obstructive pulmonary disease
ED: Emergency department
GP: General practitioner
IQR: Interquartile range
MI: Myocardial infarction
PCI: Percutaneous coronary intervention
SD: Standard deviation
SpO_2_: Peripheral oxygen saturation
TIA: Transient ischemic attack
