# Supplementary material for: Acute home-based management initiatives for COVID-19 patients needing oxygen: observational study reporting mortality and hospital admission outcomes
Source: BMC Health Serv Res. 2025 Nov 25;25:1633. doi: 10.1186/s12913-025-13733-2 (PMC12750886; doi:10.1186/s12913-025-13733-2)
Supplement: Supplementary file 1 — Supplementary Material 1 [file 12913_2025_13733_MOESM1_ESM.docx]

**Supplementary Table 1: Collaborative care initiatives**

|  | **Initiator(s)** | **Region** | **Initiative runtime** |
| --- | --- | --- | --- |
| **Initiative A** | Albert Schweitzer Hospital | Dordrecht | December 2020 – February 2022 |
| **Initiative B** | Jeroen Bosch Hospital | Den Bosch | February 2021 – June 2022 |
| **Initiative C** | Dokter Drenthe, Wilhelmina Hospital Assen | Assen | December 2021 – April 2022 |
| **Initiative D** | Julius Center for Health Sciences and Primary Care | Utrecht | November 2021 – March 2023 |
| **Initiative E** | Julius Center for Health Sciences and Primary Care | Utrecht | November 2021 – March 2023 |

**Supplementary Table 2: Patient characteristics per initiative**

|  | **Total**  n = 92 | **Initiative A**  n = 45 | **Initiative B**  n = 27 | **Initiative C**  n = 10 | **Initiative D**  n = 7 | **Initiative E**  n = 3 |
| --- | --- | --- | --- | --- | --- | --- |
| Gender male, n (%) | 50 (54.3) | 27 (60.0) | 13 (48.1) | 7 (70.0) | 1 (14.3) | 2 (66.7) |
| Age in years, mean (SD) | 62.8 (12.7) | 59.9 (12.4) | 63.6 (8.6) | 65.7 (14.0) | 77.6 (13.2) | 55.7 (23.3) |
| Age in years, min – max | 26 – 96 | 26 – 86 | 46 – 77 | 36 – 84 | 61 – 96 | 29 – 72 |
| **General health characteristics** | | | | | | |
| BMI ≥30 kg/m^2^, n (%) | 21 (33.3)  M: 29 (31.5) | 10 (34.5)  M: 16 | 7 (30.4)  M: 4 | 3 (42.9)  M: 3 | 1 (33.3)  M: 4 | 0 (0)  M: 2 |
| Current cigarette use, n (%) | 5 (6.4)  M: 14 | 2 (4.9)  M: 4 | 1 (4.5)  M: 5 | 1 (14.3)  M: 3 | 1 (16.7)  M: 1 | 0 (0)  M: 1 |
| Former cigarette use, n (%) | 24 (30.8)  M: 14 | 9 (22.0)  M: 4 | 7 (31.8)  M: 5 | 4 (57.1)  M: 3 | 4 (66.7)  M: 1 | 0 (0)  M: 1 |
| Medication use (≥ 1) n (%) | 59 (65.6)  M: 2 | 27 (60.0)  M: 0 | 18 (66.7)  M: 0 | 7 (77.8)  M: 1 | 5 (83.3)  M: 1 | 2 (66.7)  M: 0 |
| **Comorbidities** | | | | | | |
| Any comorbidities, n (%) | 43 (48.3)  M: 3 | 15 (33.3)  M: 0 | 14 (51.9)  M: 0 | 8 (100)  M: 2 | 4 (66.7)  M: 1 | 2 (66.7)  M: 0 |
| ≥2 comorbidities, n (%) | 21 (23.9)  M: 4 | 6 (13.3)  M: 0 | 6 (22.2)  M: 0 | 6 (85.7)  M: 3 | 2 (33.3)  M: 1 | 1 (33.3)  M: 0 |
| Diabetes, n (%) | 14 (15.6)  M: 2 | 6 (13.3)  M: 0 | 3 (11.1)  M: 0 | 2 (22.2)  M: 1 | 2 (33.3)  M: 1 | 1 (33.3)  M: 0 |
| COPD gold I-IV, n (%) | 11 (12.2) M: 2 | 2 (4.4)  M: 0 | 3 (11.1)  M: 0 | 2 (22.2)  M: 1 | 3 (50.0)  M: 1 | 1 (33.3)  M: 0 |
| Asthma, n (%) | 9 (10.0)  M: 2 | 4 (8.9)  M: 0 | 3 (11.0)  M: 0 | 2 (22.2)  M: 1 | 0 (0)  M: 1 | 0 (0)  M: 0 |
| Heart failure, n (%) | 3 (3.4)  M: 3 | 0 (0)  M: 0 | 1 (3.7)  M: 0 | 1 (12.5)  M: 2 | 1 (16.7)  M: 1 | 0 (0)  M: 0 |
| History of coronary disease, n (%) | 13 (14.4)  M: 2 | 5 (88.9)  M: 0 | 4 (14.8)  M: 0 | 2 (22.2)  M: 1 | 1 (16.7)  M: 1 | 1 (33.3)  M: 0 |
| Atrial fibrillation, n (%) | 7 (7.8)  M: 2 | 3 (6.7)  M: 0 | 1 (3.7)  M: 0 | 2 (22.2)  M: 1 | 1 (16.7)  M: 1 | 0 (0)  M: 0 |
| History of stroke/TIA, n (%) | 10 (11.1)  M: 2 | 3 (6.7)  M: 0 | 3 (11.1)  M: 0 | 2 (22.2)  M: 1 | 2 (33.3)  M: 1 | 0 (0)  M: 0 |
| Chronic kidney failure, n (%) | 5 (5.6)  M: 3 | 1 (2.2)  M: 0 | 2 (7.4)  M: 0 | 1 (12.5)  M: 2 | 1 (16.7)  M: 1 | 0 (0)  M: 0 |
| Immunodeficiency, n (%) | 3 (3.3)  M: 2 | 1 (2.2)  M: 0 | 1 (3.7)  M: 0 | 1 (11.1)  M: 1 | 0 (0)  M: 1 | 0 (0)  M: 0 |

Abbreviations: BMI = body mass index; CABG = coronary artery bypass grafting; COPD = chronic obstructive pulmonary disease; MI = myocardial infarction; PCI = percutaneous coronary intervention; TIA = transient ischemic attack.

**Supplementary Table 3: Clinical course data, hospital admissions and mortality per initiative**

|  | **Total**  n = 92 | **Initiative A**  n = 45 | **Initiative B**  n = 27 | **Initiative C**  n = 10 | **Initiative D**  n = 7 | **Initiative E**  n = 3 |
| --- | --- | --- | --- | --- | --- | --- |
| Home monitoring, median, min-max | 14, 1-52 | 14.5, 1-52 | 12.0, 2-45 | 15 (3-34) | 7 (1-12) | 8 (8-14) |
| Home oxygen suppletion, median, min-max | 8.5, 1-41 | 8.5, 1-26 | 10.5, 2-41 | 10, 3-18 | 4, 1-7 | 5, 1-6 |
| Hospital admissions (ward and/or ICU), n (%) | 17 (18.9)  M: 2 | 5 (11.4)  M: 1 | 8 (29.6)  M: 0 | 3 (33.3)  M: 1 | 1 (14.3)  M: 0 | 0 (0)  M: 0 |
| 30-day mortality, n (%) | 6 (6.6)  M: 1 | 1 (2.3)  M: 1 | 2 (7.4)  M: 0 | 2 (20.0)  M: 0 | 1 (14.3)  M: 0 | 0 (0)  M: 0 |

Abbreviations: ICU = intensive care unit; SD = standard deviation.
